# Older patients affected by COVID-19: investigating the existence of biological phenotypes

**DOI:** 10.1186/s12877-024-05473-5

**Published:** 2024-11-07

**Authors:** Alberto Zucchelli, Marta Parigi, Silvia Giliani, Davide Liborio Vetrano, Daniela Lucente, Emanuele Marzetti, Riccardo Calvani, Giuseppe Bellelli, Alessandra Marengoni

**Affiliations:** 1 Aging Research Center, Department of Neurobiology, Care Sciences and Society, Karolinska Institutet and Stockholm University, Stockholm, 171 77 Sweden; 2 Department of Clinical and Experimental Sciences, University of Brescia, Brescia, Italy; 3 A. Nocivelli Institute for Molecular Medicine, ASST Spedali Civili, Brescia, Italy; 4 Department of Molecular and Translational Medicine, University of Brescia, Brescia, Italy; 5 Stockholm Gerontology Research Center, Stockholm, Sweden; 6 Fondazione “Ospedale e Casa di Riposo Nobile Paolo Richiedei”, Brescia, Italy; 7 Department of Geriatrics, Orthopedics and Rheumatology, Università Cattolica del Sacro Cuore, Rome, Italy; 8 Fondazione Policlinico Universitario “Agostino Gemelli” IRCCS, Rome, Italy; 9 School of Medicine and Surgery, Milano-Bicocca University, Monza, Italy; 10 Acute Geriatric Unit, IRCCS San Gerardo Foundation, Monza, Italy

**Keywords:** Biomarkers, COVID-19, Elderly, Frailty

## Abstract

**Introduction:**

COVID-19 provides an opportunity to examine biological phenotypes (observable morphological, functional and biological characteristics) in individuals who experience the same acute condition, potentially revealing differences in response to acute external stressors. The aim our study was to investigate biological phenotypes in older patients hospitalized for COVID-19, exploiting a panel of aging biomarkers.

**Methods:**

Data were gathered from the FRACOVID Project, an observational multicenter study, aimed to evaluate the impact of frailty on health-related outcomes in patients 60 + with COVID-19 in Northern Italy. A hierarchical cluster analysis was run using log-transformed and scaled values of TNF-a, IL-1 beta, IL-6, PAI-1, GDF-15, NT-proBNP, and Cystatin C evaluated at admission.

**Results:**

Eighty-one participants (mean age 75.3 years; 60.5% male) were evaluated. Frailty was identified in 42% of the sample and 27.2% were unable to ambulate outdoors. The mean hospital stay was 24.7 days, with an in-hospital mortality rate of 18.5%. Three biological phenotypes were found: (1) ‘inflammatory’, with high inflammatory biomarkers; (2) ‘organ dysfunction’, characterized by elevated cystatin C and NT-proBNP, and lower inflammatory markers; and (3) ‘unspecific’, with lower NT-proBNP and GDF-15 levels, and intermediate concentrations of other biomarkers. The ’organ dysfunction’ phenotype showed the highest mean age and prevalence of frailty, disability, and chronic diseases. The ‘inflammatory‘ phenotype showed the highest burden of respiratory and systemic signs and symptoms of infection.

**Conclusion:**

Biological phenotypes might be used to identify different clinical and functional phenotypes in individuals affected by COVID-19.

**Supplementary Information:**

The online version contains supplementary material available at 10.1186/s12877-024-05473-5.

## Introduction

Older adults are characterized by remarkable phenotypic variability. Such variability is often described through the assessment of physical and cognitive performance, chronic illnesses, and frailty [1]. However, the aging process begins much earlier than clinical markers become explicitly evident. Changes in body composition and modifications of cellular function are considered early indicators of subsequent functional decline and the development of multimorbidity and frailty [2]. The identification and characterization of these early changes may help development early-stage, personalized diagnostic, and therapeutic interventions [3].

Recent advancements in research methodologies and technologies have facilitated the identification of biomarkers that can aid exploring the phenotypic and biological processes preceding functional alterations [4]. Inflammatory biomolecules including interleukin-6 (IL-6), tumor necrosis factor alpha (TNF-α), and other markers, such as insulin and cystatin C, have been proposed as biomarkers to be used in geroscience-guided trials [4].

This knowledge has enhanced the understanding of aging and helped conceptualize the so called “biological phenotypes”. A biological phenotype is a construct that includes observable morphological or functional traits along with molecular and biological characteristics [5]. Such constructs are receiving increased attention for their potential to facilitate the study of aging and pave the way for precision medicine in and beyond the field of geriatrics [6]. Furthermore, the concept of biological phenotypes addresses the limitations associated with the analysis of individual biomarkers by enabling the evaluation of biomarker patterns or clusters [7].

COVID-19 provides an opportunity to examine biological phenotypes in individuals who experience the same acute condition, potentially revealing biological differences in response to acute external stressors, one key element for defying frailty and biological aging [8, 9]. In this scenario, a bi-directional relationship was suggested between COVID-19 and aging, whereby specific biological hallmarks of aging (e.g., epigenetic dysregulation and telomere attrition) are associated with increased risk of SARS-CoV-2 infection and the development of severe COVID-19 [10]. In turn, COVID-19 may induce accelerated or premature aging through the stimulation of cellular senescence and the exacerbation of the senescence-associated secretory phenotype (SASP) [11]. A recent literature review examined the significance of aging biomarkers in the context of the Coronavirus Disease 2019 (COVID-19). The authors concluded that specific biomarkers could serve as predictive factors for either resistance or susceptibility to COVID-19, as well as disease severity [12].

Thus, the aim of this study was to investigate biological phenotypes in older patients who were hospitalized for COVID-19, exploiting a panel of aging biomarkers.

## Methods

### Study design and population

Data for this study were gathered from the FRACOVID Project (The effect of frailty on the clinical outcomes of patients affected by COVID-19), an observational multicenter study, aimed to evaluate the impact of frailty on adverse health-related outcomes in middle-aged and older individuals hospitalized for COVID-19 [13]. The study protocol obtained ethical approval from the Brianza Institutional Review Board (approval code 3356-07/08/2020) and was registered in clinicaltrials.gov (NCT04412265). All patients, or their proxies as needed, gave oral consent for participation in the study at ward admission.

The study population was composed of consecutive patients with COVID-19 hospitalized from 27 February 2020 to 4 May 2020 in the acute Geriatric and Infectious disease wards of the San Gerardo Hospital (Monza, Italy) and the Civili Hospital (Brescia, Italy). Only participants older than 18 years, with a diagnosis of COVID-19 from a positive polymerase chain reaction test on SARS-CoV-2 nasopharyngeal swab, and with a Clinical Frailty Scale (CFS) score of 7 or lower were included. Considering the increased clinical workload prompted by the COVID-19 emergency during the research period, only a random subsample of participants enrolled in Monza were selected for blood sampling and subsequent biomarker analysis. For this study we have included only the participants older than 60 years old within this subsample.

### Data collection

Data collection was performed by trained personnel using a structured case report form (CRF) and an online Research Electronic Data Capture (REDCap) platform. Personal and clinical information was collected via in-person interviews (or phone interviews with patients’ proxies), medical examinations, and a careful review of medical records. Data included sociodemographic characteristics, smoking habits, date of onset of COVID-19 signs/symptoms, functional status, chronic diseases, prescribed drugs, health status at ward admission (vital signs, need for oxygen therapy, and results from biochemical analyses and radiological examination) and during hospitalization (prescribed therapy, need for transfer to a higher intensity care unit, survival status).

We considered a range of chronic conditions, including cardiac diseases (ischemic or valvular), atrial fibrillation, stroke, chronic kidney disease, and chronic obstructive pulmonary disease (COPD). Additionally, we examined three geriatric syndromes: malnutrition, dementia, and frailty. Malnutrition was assessed using the Body Mass Index (BMI), using a value < 18 as cutoff. Dementia was evaluated based on prior diagnoses or the use of specific medications. The frailty status prior to SARS-CoV-2 infection was assessed at hospital admission by a geriatrician using the CFS. The CFS is an ordinal 9-point scale in which the assessor makes decisions about the degree of frailty from clinical data [14]. Individuals are scored from 1 “very fit” to 9 “terminally ill”, with a score between 6 and 8 being indicative of moderate to severe frailty. The CFS was further used to categorized the study participants as frail (CFS ≥ 5) or non-frail (CFS < 5). Disability status was considered as having lost autonomy in at least one instrumental (IADL) or basic activities of daily living. A chest X-ray at admission was considered positive multiple consolidations were present. The Brescia-COVID Respiratory Severity Scale [15] was used to evaluate COVID-19 severity through the assessment of clinical signs of distress and oxygen need.

Total white blood cell count (WBC), absolute number of lymphocytes, creatinine, and C-reactive protein concentration at admission were also collected as part of the routine blood examination panel.

### Analysis of biomarkers

Serum/Plasma were collected by centrifugation at 3000 rpm for 10 min, aliquoted, and stored at − 80 °C.

until analysis. Samples were processed on Luminex MAGPIX^®^ (EMD Millipore^®^) according to the manufacturer’s instructions using ProcartaPlex Mix&Match 8-plex (GDF-15, IL-1 beta, IL-6, MMP-1, NTproBNP, PAI-1, TNF alpha) and ProcartaPlex Human Cystatin C Simplex Bead Panel (Thermo Fisher). Before conducting the present analyses, we performed a preliminary investigation of the dynamic range of the MAGPIX^®^ system using serum samples from older patients. The resulting calibration curves demonstrated robust discrimination. All analyses presented in this study were conducted in duplicate, with no significant deviation observed between the two sets of data.

The data were analyzed using ProcartaPlex Analysis App (Thermo Fisher).

The panel of geroscience markers included:

- Cystatin C: a molecule produced by all nucleated cells, shown to reflect renal function and to be strongly associated with mortality, in particular in older adults [16], and unsuccessful aging [17].
- Growth differentiation factor 15 (GDF-15), a divergent member of the transforming growth factor beta cytokines family, which is upregulated in the setting on tissue injury, inflammation, hypoxia, and oxidative stress [18]. GDF-15 has been associated with mitochondrial dysfunction and decreased survival in old age [19].
- IL-1β, a pro-inflammatory cytokine considered one of the most relevant markers of inflammaging [7].
- IL-6, a pro-inflammatory cytokine that has been proposed as one of the first biomarkers of aging [20] and is associated with frailty [21] and chronic diseases [20].
- N-terminal pro-B-type natriuretic peptide (NT-proBNP), a biomarker associated with cardiac health, widely used in the diagnosis and prognostication of heart failure [22]. NT-proBNP has also been associated with aging all-cause mortality [23].
- Plasminogen activator inhibitor-1 (PAI-1), a serine protease inhibitor that inhibits tissue-type plasminogen activator and urokinase. It has been associated with myocardial infraction, aortic valve stenosis as well as with aging and cellular senescence [24].
- TNF-a, a pro-inflammatory cytokine that has been associate with atherosclerosis, inflammaging [25], frailty, sarcopenia [26].

### Statistical analysis

We conducted a cluster analysis using the panel of biomarkers available. It has been suggested that clustering analysis and other machine-learning techniques might help to better understand the biological profile of study participants, in comparison with simpler approaches such as regressions [7].

Biomarker data that were out of range were removed from the analysis. After log-transformation and visual evaluation of approximate normal distribution, biomarker values were centered and scaled using mean and standard deviation, respectively. Study participants were grouped according to hierarchical clustering, using Ward’s method and squared Euclidean distance, as previously described [27].

The visual inspection of the resulting dendrogram (Figure S1) was used to select the appropriate number of clusters. Participants’ characteristics were described using mean and standard deviation (SD), count and proportion, or median and interquartile range (IQR), as appropriate. Differences in personal, clinical, and functional characteristics among clusters were evaluated using one-way ANOVA, chi-square tests, or Fisher’s exact tests, as appropriate. A two-tailed p value < 0.05 was considered statistically significant. The medians of biomarkers’ concentrations across the different biological phenotypes were compared using the Kruskal-Wallis test. All analyses were conducted using R 4.3.0 [28].

## Results

A total of 93 blood samples were collected from participants aged over 60 years. Twelve individuals were excluded due to out-of-range IL-6b (*N* = 9) or IL-6 (*N* = 3) values, resulting in 81 participants for analysis. Those excluded exhibited a similar age, CFS score and disability prevalence, although the concentration of CRP seemed to be lower in comparison to those included, as shown in Table S2. As shown in Table 1, the average age of the cohort was 75.3 years (Standard Deviation - SD: 10.9 years), with 60.5% (49 participants) being male. Frailty was identified in 42% (34 participants), and 27.2% were unable to ambulate outdoors. The most prevalent chronic conditions included cardiovascular diseases (33.3%), malnutrition (25.9%), and dementia (19.8%). At hospital admission, a respiratory rate above 20 breaths per minute was observed in 39.5% of participants, and 30.9% presented with fever. According to the BRESCIA COVID scale, 17.3% were breathing ambient air, 67.9% required supplemental oxygen, and 14.8% were on non-invasive ventilation. The mean hospital stay was 24.7 days (SD: 12.7), with an in-hospital mortality rate of 18.5%.

**Table 1:** Sociodemographic and clinical characteristics of study participants according to biomarker patterns

		Biomarker patterns			
Mean (SD) or N (%)	Overall	Inflammatory *N* = 33 (40.7)	Organ dysfunction *N* = 30 (37.1)	Unspecific *N* = 18 (22.0)	p
Age (years), mean (SD)	75.3 (10.9)	75.7 (8.3)	79.8 (12.0)^b^	66.9 (8.5)	< 0.001
Male sex, n (%)	49 (60.5)	17 (51.5)	19 (63.3)	13 (72.2)	0.325
Living alone, n (%)	35 (50.0)	14 (50.0)	15 (51.7)	6 (46.2)	0.946
Disability (any ADL or IADL lost), n (%)	36 (44.0)	13 (39.4)	19 (63.3)^b^	4 (22.2)	0.016
Clinical Frailty Scale	4.2 (2.0)	3.7 (1.8)	5.3 (1.7)^a, b^	3.2 (1.9)	< 0.001
Clinical Frailty Scale ≥ 5	34 (42.0)	11 (33.3)	19 (63.3)^a, b^	4 (22.2)	0.010
Prescription drugs, n (%)	5.1 (3.6)	5.0 (4.0)	6.4 (3.3)^b^	3.4 (2.6)	0.018
Time from symptoms onset and hospital admittance (days), mean (SD)	9.5 (8.2)	10.2 (9.8)	8.5 (7.4)	9.9 (6.4)	0.711
Hospital LOS (days), mean (SD)	24.7 (12.7)	24.4 (11.0)	28.3 (16.2)	20 (8.0)	0.450
In-hospital mortality, n (%)	15 (18.5)	6 (18.2)	7 (23.3)	2 (11.1)	0.621
**Chronic conditions**					
Malnutrition, n (%)	21 (25.9)	5 (15.2)	13 (43.3)^a^	3 (16.7)	0.029
Heart disease, n (%)	27 (33.3)	11 (33.3)	13 (43.3)	3 (16.7)	0.165
Stroke, n (%)	7 (8.6)	2 (6.1)	4 (13.3)	1 (5.6)	0.594
Dementia, n (%)	16 (19.8)	5 (15.2)	9 (30.0)	2 (11.1)	0.267
Chronic kidney disease, n (%)	9 (11.1)	0 (0.0)	9 (30.0)^a, b^	0 (0.0)	< 0.001
Solid tumor, n (%)	9 (11.1)	6 (18.2)	1 (3.3)	2 (11.1)	0.165
COPD, n (%)	6 (7.4)	4 (12.1)	2 (6.7)	0 (0.0)	0.364
**Characteristics at hospital admission**					
Respiratory rate ≥ 20/min, n (%)	32 (39.5)	18 (54.5)	11 (36.7)	3 (16.7)	0.028
Heart rate ≥ 100/min, n (%)	18 (22.2)	9 (27.3)	4 (13.3)	5 (27.8)	0.336
Fever, n (%)	25 (30.9)	12 (36.4)	7 (23.3)	6 (33.3)	0.518
Positive chest x-ray, n (%)	69 (85.2)	31 (93.9)	23 (76.7)	15 (83.3)	0.151
Brescia COVID scale					0.022
Ambient air, n (%)	14 (17.3)	6 (18.2)	7 (23.3)^a, b^	1 (5.6)	
Oxygen support, n (%)	31 (38.3)	9 (27.3)	13 (43.3)^a^	9 (50.0)	
Oxygen support, distressed patient, n (%)	24 (29.6)	11 (33.3)	10 (33.3)	3 (16.7)	
Continuous Positive Air Pressure ventilation, n (%)	12 (14.8)	7 (21.2)	0 (0.0)^a, b^	5 (27.8)	
White blood cells (10^3^/mL), mean (SD)	7.3 (3.2)	7.3 (3.0)	7.4 (3.3)	7.0 (3.3)	0.918
C-reactive protein (mg/mL), mean (SD)	8.3 (6.8)	8.2 (5.7)	7.5 (7.9)	9.7 (6.9)	0.595
Lymphocytes (10^3^/mL, mean (SD)	1.2 (0.5)	1.1 (0.5)	1.3 (0.6)	1.2 (0.6)	0.625
Creatinine (mg/mL), mean (SD)	1.4 (1.5)	1.0 (0.3)	2.1 (2.3)^a, b^	0.9 (0.2)	0.004

*Abbreviations* LOS = length of stay; ADL = activities of daily living; IADL = instrumental activities of daily living; COPD = chronic obstructive pulmonary disease; mRASS = modified Richmond agitation and sedation scale. Missing = 22 for hospital LOS, 9 for living alone, 4 for Time from symptoms onset and hospital admittance. a = *p* < 0.05 vs. ‘inflammatory’, b = *p* < 0.05 vs. ‘unspecific’

**Fig. 1:**
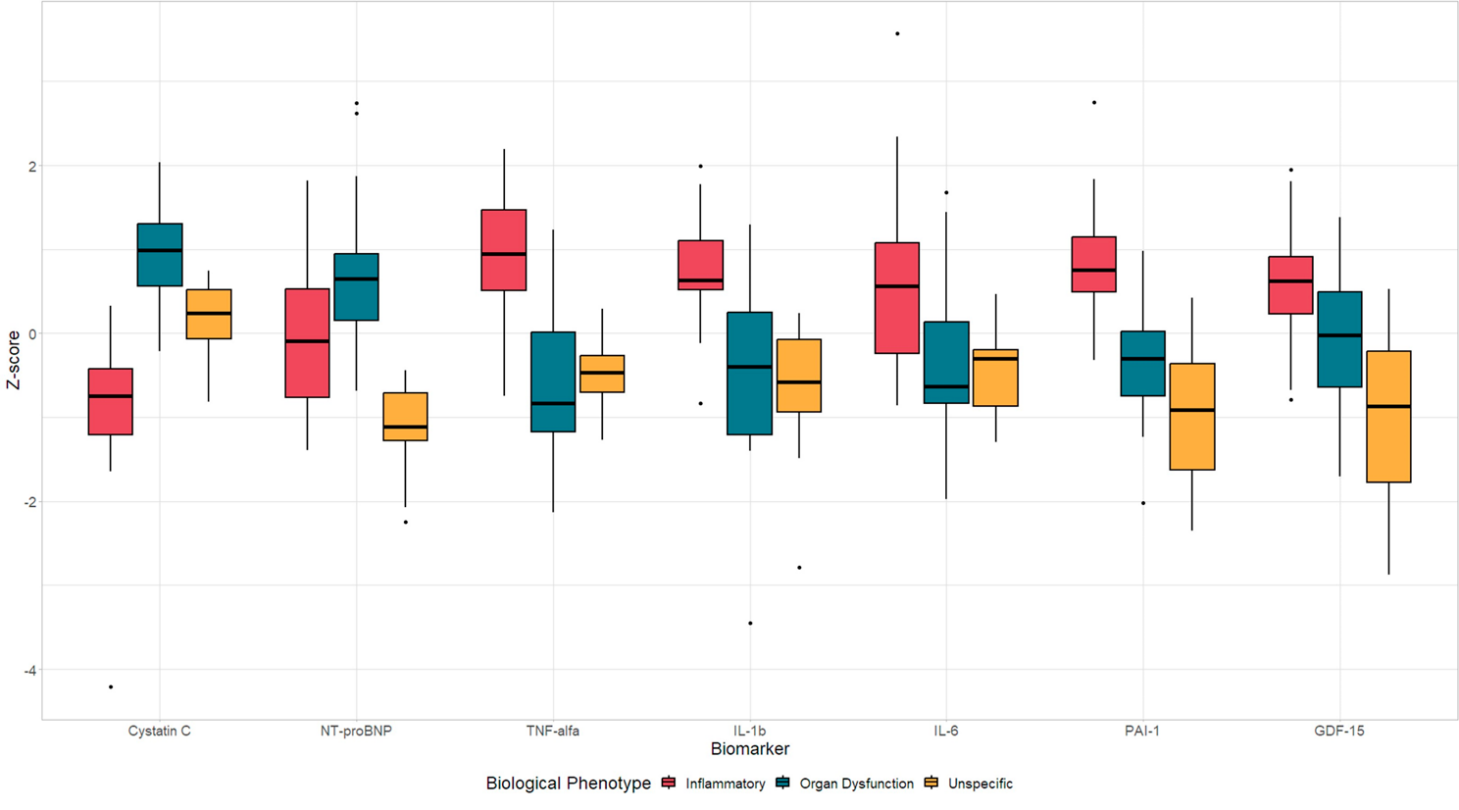
Boxplot showing the distribution of all biomarkers evaluated (shown as Z-scores) across the three biological phenotypes

Cluster analysis revealed three distinct biological phenotypes (Fig. 1 and Table S1): (1) ‘inflammatory’, with high inflammatory biomarkers; (2) ‘organ dysfunction’, characterized by elevated cystatin C and NT-proBNP, and lower inflammatory markers; and (3) ‘unspecific’, with lower NT-proBNP and GDF-15 levels, and intermediate concentrations of other biomarkers.

The participants assigned to the ‘organ dysfunction’ biological phenotype exhibited the highest mean age (79.8 years old), mean CFS score (5.3) and mean number of chronically prescribed drugs (6.4), as well as the highest proportion of frailty (63.3%), disability (63.3%), and in-hospital mortality (23.3%). This group also showed the highest prevalence of malnutrition (43.3%), heart diseases (43.3%), stroke (13.3%), dementia (30.0%), and chronic kidney disease (30.0%). At admission, the 23.3% of the participants in this group had fever and 76.6% of them need an oxygen supplementation, although none of the patients in this group was ventilated.

Those assigned to the ‘inflammatory’ biological phenotype had a mean age of 75.7 years old and a mean CFS score of 3.7. One third of the patients in this group was affected by frailty and the 39.4% lived with disability. This phenotype showed the highest proportion of COPD (12.1%) and solid tumour (18.2%). In total, the 81.8% of the participants within this phenotype needed oxygen supplementation at admission (25.9% of them was non-invasively ventilated). In-hospital mortality was 18.2%.

The lowest mean age was shown by the participants assigned to the ‘unspecific’ biological phenotype (66.9 years old). This group also showed the lowest mean CFS score (3.2) and the lowest prevalence of frailty (22.2%) and disability (22.2%). The participants clustered in this group showed the lowest in-hospital mortality (11.1%) and the highest proportion of oxygen supplementation at admission (94.4%).

The participants included in the ‘inflammatory’cluster, compared to those classified in the ‘organ dysfunction’ cluster, showed a significantly lower frailty, prevalence of malnutrition and chronic kidney disease, as well as a higher Brescia COVID scale (all *p* < 0.05).

## Discussion

The findings from the present study show that, in older patients hospitalized for COVID-19, specific biological signatures are associated with different clinical/functional phenotypes. Three biological phenotypes were identified, one characterized by high levels of inflammation, one with high concentrations of cystatin C and NT-proBNP and lower inflammation, and one exhibiting intermediate concentration of most biomarkers. This latter ‘unspecific’ biological phenotype was characterized by the lowest mean age and the lowest frequency of disability and frailty. Conversely, participants clustered in the ‘organ dysfunction’ phenotype exhibited the highest mean CFS score and the highest proportion of functional dependence.

Our study’s design, focusing on biomarkers during an acute condition, limits our ability to determine whether these biomarker concentrations are altered due to pre-existing conditions, acute inflammatory status, or a combination thereof. However, some considerations are worth discussing.

The biological phenotypes identified in our study may reflect individual responses to an acute infectious disease. Specifically, in the context of COVID-19, a robust inflammatory response is present, as evidenced by elevated levels of IL-1 beta, IL-6, and TNF-alpha [10, 11]. These cytokines are not only heightened in COVID-19 patients but also serve as predictors of disease severity [29, 30]. The overproduction of these inflammatory cytokines during COVID-19 serves also as the main rationale for the use of specific drugs (such Tocilizumab, Sarilumab, and Anakinra) in the treatment of severe forms of the disease [30–32]. Even if the concentration of C-Reactive Protein and the total number of leukocytes and lymphocytes did not significantly differ between phenotypes, the subgroup presenting with the ‘inflammatory’ phenotype exhibited a high prevalence of symptoms and signs such as tachypnea, tachycardia, fever, radiographic chest abnormalities, and requirement for oxygen supplementation, suggesting a pronounced systemic inflammatory response to the infection. Conversely, the ‘unspecific’ phenotype showed lower inflammatory cytokine levels (comparable to the ‘organ dysfunction’ group), but their clinical presentation at hospital admission resembled the ‘inflammatory’ phenotype. The higher mortality shown by the ‘inflammatory’ group, in comparison with the ‘unspecific’ phenotype might again corroborate the hypothesis of an excessive inflammatory response in the former phenotype. However, the influence of factors such as age, frailty, and disability on mortality rates cannot be overlooked, given the higher prevalence of these conditions among participants in the ‘inflammatory’ biological phenotype in comparison with the ‘unspecific’ one. Indeed, the majority of those included in the ‘unspecific’ phenotype were participants younger than 70 years old and exhibited functional characteristics that are more typical of younger and healthier older adults: future studies, with a larger sample size are warranted to further discriminate the relationship between age, clinical frailty and biological phenotypes. Furthermore, it is also noteworthy that the ‘inflammatory’ phenotype had a higher prevalence of COPD and solid tumors, conditions that typically induce a pro-inflammatory state [33, 34]: whether the elevated inflammatory biomarkers in this group are solely attributable to the acute COVID-19 response or a combination of basal inflammation and acute infection remains unclear in our data. The ‘organ dysfunction’ phenotype’s clinical profile aligns with existing literature on older, frailer individuals with chronic conditions, who often display atypical symptoms and disease progression, yet facing high mortality rates [9, 35], probably due to a pervasive immunosenescence [36]. This latter condition is likely to be attributable for the low concentration of inflammatory biomarkers found in this phenotype. Conversely, the high concentrations of cystatin C and NT-proBNP in these phenotype might be a proxy of the loss of physiological reserve of several organs and systems, in line with previous studies [16, 23]. We cannot conclude on whether the damage of organs and system is due to pre-existing conditions (as suggested by the high burden of chronic diseases found in this biological phenotype), to the role of the SARS-CoV-2 infection or a combination of the two. Our data confirm that in the context of COVID-19 persons with frailty exhibited high mortality rates [37, 38], suggesting, however, that this may happen even in absence of an extremely high concentrations of inflammatory cytokines. Due to the study design, we cannot exclude the possibility that the concentration of inflammatory cytokines may be partially explained by a chronic condition of low-grade inflammation, known as inflammaging, which has already been suggested to be associated with poorer outcomes in older persons with SARS-CoV-2 infection [39, 40].

The evaluation of biological signatures in the context of acute conditions may be useful to differentiate patients who are likely to benefit to some therapeutic and preventative approaches, such as antivirals or immunomodulatory, from those who are likely to exhibit a poor response. Indeed, several studies showed conflicting results in terms of efficacy of various anti-inflammatory drugs [41–44]. Characterizing the biological profile (either pre-existing or modified by SARS-CoV-2 infection) may aid in better understanding the role of these drugs in individuals with certain phenotypes. In addition, such information could be used to define the characteristics of participants in randomized clinical trials on new drugs and vaccines, and to prioritize vaccination campaign or decide the number of vaccine shots needed. Notably, findings of this study may have a high potential translational use in other infectious diseases.

Other studies previously attempted to cluster individuals with COVID-19 using biomarkers. A study identified different clusters of patients affected by COVID-19 using information such as demographic characteristics, co-morbidities and clinical presentation [45]. Among the 5 clusters identified among hospitalized patients, one cluster was characterized by older age (median age 71), a high concentration of C-Reactive Protein (median 9 mg/dL) and an in-hospital mortality rate of 20%, probably overlapping with the ‘inflammatory’ biological phenotype highlighted in our study. A cluster of older patients affected by COVID-19 characterized by high levels of CRP and high mortality rate was also reported by the study of Cidade JP et al. [46]. Using several inflammatory biomarkers drawn from 129 patients with COVID-19 and topological data analysis, Blair PW et al. [47]identified 3 clusters: the cluster exhibiting the highest concentration of inflammatory biomarkers, in particular IL-1RA, was also characterized by the highest age (median 51.8) and the highest mortality and hospitalization rate.

Among the considered inflammatory biomarkers, GDF-15 and PAI-1 deserve attention. GDF-15 is considered a biomarker of mitochondrial dysfunction and a marker of biological aging [4]. However, the actual significance of GDF-15 is still debated with evidence reporting opposite functions, with some studies showing an increment during an acute stress response [7, 48].PAI-1 is a SERPIN inhibitor, primarily known for its regulation of fibrinolysis, but also causally associated with several aging-related chronic diseases and a marker of senescence [49]. PAI-1 is also a well-recognized acute phase protein and there is emerging evidence regarding its central role in COVID-19-associated endothelial dysfunction, supporting PAI-1 as a potential mechanistic link between known risk factors (e.g., tobacco use, age) and clinical manifestations of COVID-19 [50]. PAI-1 has also been associated with some chronic heart conditions [24], possibly explaining why the concentrations of this biomarkers were found to be higher in the ‘organ dysfunction’ phenotype in comparison with the ‘unspecific’ one.

### Strengths and limitations

In this study we have evaluated both biomarkers of aging and functional measure, as well as frailty in the context of COVID-19 pandemic, offering a multidimensional appraisal of the characteristics of older patients hospitalized with an acute condition. Although limited by the challenges posed by the pandemic, the functional and clinical characteristics of the study population were collected by geriatricians and resident in geriatrics, assuring the quality of the information collected. We used an unsupervised statistical approach that allowed us to simultaneously evaluate multiple biomarkers, an approach that may overcome the limitations posed by the evaluation of single biomarkers.

The results of our study should be interpreted in light of some limitations. First, the sample size was limited. Although some trends are already detectable in our analysis, further studies with a larger population are needed to better identify biomarker patterns. In particular, future studies may help to better characterize the differences between the ‘inflammatory’ and the ‘organ dysfunction’ phenotypes, helping to better identify the clinical, function, and socio-demographic characteristics of patients included in such clusters. Second, no information was available on the biomarker profiles prior to the infection by SARS-CoV-2, which hampers the possibility to establish causal links between biological phenotypes, functional characteristics, COVID-19, and clinical outcomes. Third, the data were collected in a single centre and in a limited time window, possibly limiting the generalizability of our results. Fourth, despite the data were collected in a restricted timeframe and similar durations from symptom onset to hospital admission across phenotypes, variations in infection timing or differences in the SARS-CoV-2 strain cannot be entirely ruled out.

## Conclusion

Biological phenotypes, might be used to identify different clinical and functional phenotypes, in individuals affected by COVID-19. The ‘inflammatory’, ‘organ dysfunction’, and ‘unspecific’ biological phenotypes differed by demographic and functional characteristics, highlighting the need for further studies aimed at identifying individualized diagnostic and therapeutic pathways, even for patients hospitalized with the same acute condition.

## Electronic supplementary material

Below is the link to the electronic supplementary material.

Supplementary Material 1

Supplementary Material 2

## Data Availability

The datasets used and analysed during the current study available from the corresponding author on reasonable request.
