# Supplementary material for: Older patients affected by COVID-19: investigating the existence of biological phenotypes
Source: BMC Geriatr. 2024 Nov 7;24:923. doi: 10.1186/s12877-024-05473-5 (PMC11542346; doi:10.1186/s12877-024-05473-5)

**Table S1**. Details the investigated biomarker values in pg/mL (median [IQR]).

| UI/L | Overall | Inflammatory | Organ dysfunction | Unspecific | *p* |
| --- | --- | --- | --- | --- | --- |
| Cystatin C | 383,180.6 [257,346.1, 580,833.4] | 230,732.1 [170,940.1, 284,222.0] | 711,335.3 [539,165.1, 872,294.8] | 437,454.5 [358,268.1, 523,244.0] | <0.001 |
| GDF-15 | 1,037.4 [619.6, 1597.6] | 1,524.7 [1,137.2, 1,888.8] | 939.1 [598.9, 1,380.1] | 508.1 [255.3, 815.3] | <0.001 |
| IL-1b | 5.8 [2.6, 8.1] | 8.5 [7.7, 12.7] | 3.4 [1.7, 6.0] | 2.9 [2.2, 4.5] | <0.001 |
| IL-6 | 25.1 [14.0, 68.6] | 67.0 [24.6, 128.3] | 15.1 [11.8, 39.4] | 22.7 [11.4, 26.1] | <0.001 |
| NT-proBNP | 53.6 [18.9, 171.7] | 49.2 [17.8, 126.6] | 152.2 [71.3, 237.4] | 10.6 [8.3, 19.4] | <0.001 |
| PAI-1 | 908.8 [591.6, 1,351.0] | 1,412.1 [1,205.7, 1,798.7] | 743.6 [565.9, 906.0] | 512.8 [332.1, 715.1] | <0.001 |
| TNF-a | 6.7 [4.6, 12.7] | 13.9 [10.4, 19.6] | 4.3 [3.5, 7.5] | 5.5 [4.7, 6.2] | <0.001 |

**Figure S1** Dendogram of the hierarchical cluster analysis.


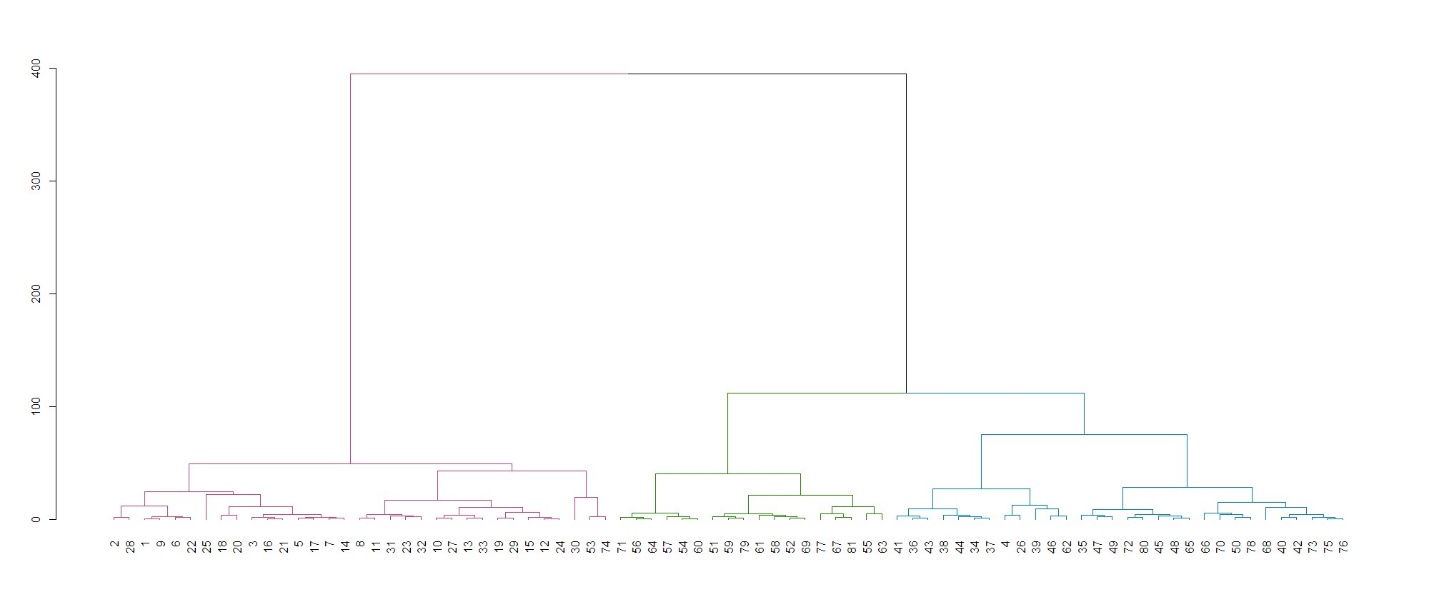

Supplement: Supplementary file 1 — Supplementary Material 1 [file 12877_2024_5473_MOESM1_ESM.docx]
