# Supplementary material for: Older patients affected by COVID-19: investigating the existence of biological phenotypes
Source: BMC Geriatr. 2024 Nov 7;24:923. doi: 10.1186/s12877-024-05473-5 (PMC11542346; doi:10.1186/s12877-024-05473-5)
Supplement: Supplementary file 2 — Supplementary Material 2 [file 12877_2024_5473_MOESM2_ESM.docx]

|  | | | Biomarker patterns | | |  |  |
| --- | --- | --- | --- | --- | --- | --- | --- |
| Mean (SD) or N (%) | | Overall N = 81 | Inflammatory N = 33 (40.7) | Organ dysfunction  N = 30 (37.1) | Unspecific  N = 18 (22.0) | **EXCLUDED N = 12** | p |
| Age (years), mean (SD) | | 75.3 (10.9) | 75.7 (8.3) | 79.8 (12.0) | 66.9 (8.5) | 76.3 (10.4) | 0.768 |
| Male sex, n (%) | | 49 (60.5) | 17 (51.5) | 19 (63.3) | 13 (72.2) | 7 (58.3) | 1.000 |
| Living alone, n (%) | | 35 (50.0) | 14 (50.0) | 15 (51.7) | 6 (46.2) | 3 (33.3) | 0.484 |
| Disability (any ADL or IADL lost), n (%) | | 36 (44.0) | 13 (39.4) | 19 (63.3) | 4 (22.2) | 6 (50.0) | 1.000 |
| Clinical Frailty Scale | | 4.2 (2.0) | 3.7 (1.8) | 5.3 (1.7) | 3.2 (1.9) | 4.5 (2.2) | 0.613 |
| Clinical Frailty Scale ≥ 5 | | 34 (42.0) | 11 (33.3) | 19 (63.3) | 4 (22.2) | 7 (58.3) | 0.357 |
| Prescription drugs, n (%) | | 5.1 (3.6) | 5.0 (4.0) | 6.4 (3.3) | 3.4 (2.6) | 3.3 (3.4) | 0.107 |
| Time from symptoms onset and hospital admittance (days), mean (SD) | | 9.5 (8.2) | 10.2 (9.8) | 8.5 (7.4) | 9.9 (6.4) | 21.9 (15.4) | 0.019 |
| Hospital LOS (days), mean (SD) | | 24.7 (12.7) | 24.4 (11.0) | 28.3 (16.2) | 20 (8.0) | 20.6 (11.3) | 0.294 |
| In-hospital mortality, n (%) | | 15 (18.5) | 6 (18.2) | 7 (23.3) | 2 (11.1) | 1 (8.3) | 0.684 |
|  | **Chronic conditions** | | | | | | |
| Malnutrition, n (%) | | 21 (25.9) | 5 (15.2) | 13 (43.3) | 3 (16.7) | 2 (16.7) | 0.724 |
| Heart disease, n (%) | | 27 (33.3) | 11 (33.3) | 13 (43.3) | 3 (16.7) | 3 (25.0) | 1.000 |
| Stroke, n (%) | | 7 (8.6) | 2 (6.1) | 4 (13.3) | 1 (5.6) | 1 (8.3) | 1.000 |
| Dementia, n (%) | | 16 (19.8) | 5 (15.2) | 9 (30.0) | 2 (11.1) | 4 (33.3) | 0.280 |
| Chronic kidney disease, n (%) | | 9 (11.1) | 0 (0.0) | 9 (30.0) | 0 (0.0) | 0.0 (0.0) | 0.599 |
| Solid tumor, n (%) | | 9 (11.1) | 6 (18.2) | 1 (3.3) | 2 (11.1) | 1 (8.3) | 1.000 |
| COPD, n (%) | | 6 (7.4) | 4 (12.1) | 2 (6.7) | 0 (0.0) | 1 (8.3) | 1.000 |
|  | **Characteristics at hospital admission** | | | | | | |
| Respiratory rate ≥ 20/min, n (%) | | 32 (39.5) | 18 (54.5) | 11 (36.7) | 3 (16.7) | 3 (25.0) | 0.525 |
| Heart rate ≥ 100/min, n (%) | | 18 (22.2) | 9 (27.3) | 4 (13.3) | 5 (27.8) | 1 (8.3) | 0.448 |
| Fever, n (%) | | 25 (30.9) | 12 (36.4) | 7 (23.3) | 6 (33.3) | 0 (0.0) | 0.032 |
| Positive chest x-ray, n (%) | | 69 (85.2) | 31 (93.9) | 23 (76.7) | 15 (83.3) | 7 (58.3) | 0.040 |
| Brescia COVID scale | |  | | | |  | 0.092 |
| Ambient air, n (%) | | 14 (17.3) | 6 (18.2) | 7 (23.3) | 1 (5.6) | 6 (50.0) |  |
| Oxygen support, n (%) | | 31 (38.3) | 9 (27.3) | 13 (43.3) | 9 (50.0) | 4 (33.3) |  |
| Oxygen support, distressed patient, n (%) | | 24 (29.6) | 11 (33.3) | 10 (33.3) | 3 (16.7) | 1 (8.3) |  |
| Continuous Positive Air Pressure ventilation, n (%) | | 12 (14.8) | 7 (21.2) | 0 (0.0) | 5 (27.8) | 1 (8.3) |  |
| White blood cells (10^3^/mL), mean (SD) | | 7.3 (3.2) | 7.3 (3.0) | 7.4 (3.3) | 7.0 (3.3) | 8.6 (4.7) | 0.212 |
| C-reactive protein (mg/mL), mean (SD) | | 8.3 (6.8) | 8.2 (5.7) | 7.5 (7.9) | 9.7 (6.9) | 2.8 (2.0) | 0.007 |
| Lymphocytes (10^3^/mL , mean (SD) | | 1.2 (0.5) | 1.1 (0.5) | 1.3 (0.6) | 1.2 (0.6) | 1.7 (1.6) | 0.036 |
| Creatinine (mg/mL), mean (SD) | | 1.4 (1.5) | 1.0 (0.3) | 2.1 (2.3) | 0.9 (0.2) | 0.8 (0.2) | 0.186 |

*p value* refers to the difference between “overall” (included participants) and excluded participants due to “out of range” values in somebiomarkers
